# Band of cacophony - abdominal catastrophe caused by the fibrous band of Meckel’s diverticulum: a case report

**DOI:** 10.4076/1757-1626-2-7160

**Published:** 2009-07-27

**Authors:** Gautam Dutta, Aparna Saha Chowdhury, Mukta Panda

**Affiliations:** Department of Internal Medicine, University of Tennessee College of MedicineChattanooga 960 East Third Street, Suite 208, TN 37403USA

## Abstract

**Introduction:**

This report calls attention to small bowel necrosis resulting as a complication of formation of an obstructive loop of small bowel around a band of Meckel’s diverticulum.

**Case presentation:**

We report a case of an adult in his 5th decade presenting with sharp, colicky abdominal pain. On presentation his vitals were within normal limits, abdomen was non-distended but tender and rigid all over, more on left lower quadrant without any rebound tenderness. Bowel sounds were hypoactive. Rectal exam showed an empty vault. White blood cell count was 9.0 x 10^3^/mm^3^ with 94.5% neutrophils, Hb of 9.0 gm/dl and Hct of 31.3%, liver and pancreatic enzymes were not elevated. Arterial blood gas did not show any acidosis and lactic acid level was not elevated. X-ray showed a non-obstructive bowel pattern without any free air. Abdominal computed tomography with oral and intravenous gastrograffin showed findings consistent with complete mid to distal small bowel obstruction secondary to a closed loop obstruction. Emergent laparotomy showed a Meckel's diverticulum that had formed a band around a portion of small bowel causing it to twist upon itself and become necrotic.

**Conclusion:**

Histopathology revealed Meckel’s diverticulum and benign intestinal tissue with hemorrhagic necrosis.

## Introduction

Meckel’s diverticulum (MD) is an ileal diverticulum located 100 cm proximal to the cecum. It results from failure of the omphalomesenteric duct to obliterate completely. This failure can then lead to multiple anatomical problems: omphalomesenteric fistula, an enterocyst, a fibrous band connecting the intestine to the umbilicus or a Meckel’s diverticulum with or without a fibrous band connecting to the umbilicus [1]. Meckel’s diverticulum contains all layers of the intestinal wall and has its own mesentery and blood supply (branch of the superior mesenteric artery). The majority of complicated cases of MD contain ectopic mucosa (75% gastric, 15% pancreatic) [1-3]. This leads to ulceration and bleeding of ileal mucosa adjacent to the acidic ectopic gastric secretions. Alkaline secretions of ectopic pancreatic tissue can also cause ulcerations.

Meckel’s diverticulum, based on autopsy studies and intraoperative evidence, occurs in 0.3% to 4% of the population and is the most prevalent congenital anomaly of the gastrointestinal tract [1]. Complications occur more frequently in males. Most patients who develop symptoms are younger than 10 years. While bleeding is the most common complication in children, intestinal obstruction seems to be the most common complication in adult age group [1]. Despite its rarity, MD should always be considered in the differential diagnosis of or unexplained acute or intermittent abdominal pain, nausea and vomiting, rectal bleeding, peritonitis, or obstruction in the older age group because it can cause significant mortality and morbidity.

## Case presentation

**Figure 1.**
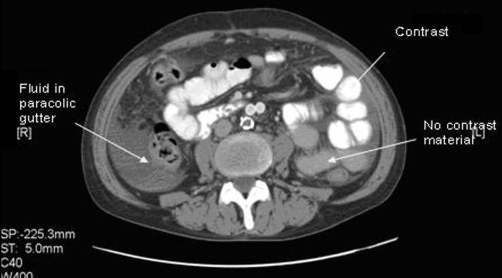
CT abdomen with and without contrast showing differential contrast distribution.

**Figure 2.**
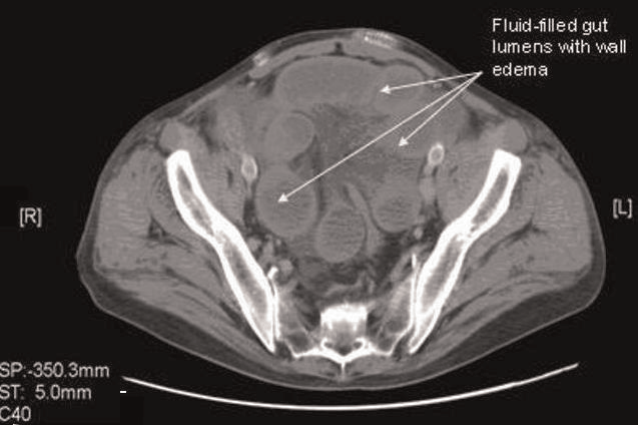
CT abdomen with and without contrast showing fluid-filled gut lumens with wall edema.

A 55-year-old white male presented with progressively worsening abdominal pain for 3 days. The pain started in the mid-lower abdomen and then became generalized, sharp and colicky, 10/10 in intensity, non-radiating, associated with nausea but no vomiting. Though he had noticed blood in his stools occasionally in the past, his last bowel movement was three days prior to presentation with semisolid stool without any blood. His past medical history was significant for hypertension and peripheral vascular disease, status post aorto-femoral bypass graft 6 years prior. On presentation his vital signs were within normal limits, abdomen was non-distended but diffusely tender and rigid, more on left lower quadrant without any rebound tenderness. Bowel sounds were hypoactive. Rectal exam showed an empty vault. WBC count was 9.0 x 103 / mm^3^ with 94.5 % neutrophils, hemoglobin of 9.0 gm/dl and hematocrit of 31.3 %, liver and pancreatic enzymes were not elevated. ABG didn’t show any acidosis and lactic acid level was not elevated. X-ray showed a non-obstructive bowel pattern without any free air. Abdominal CT with oral and IV gastrograffin showed findings consistent with complete mid to distal small bowel obstruction secondary to a closed loop obstruction (Figures 1, 2). Emergent laparotomy showed a MD that had formed a band around a portion of small bowel causing it to twist upon itself with subsequent necrosis. Histopathology showed MD with hemorrhagic necrosis and benign intestinal tissue with necrosis.

## Discussion

**Figure 3.**
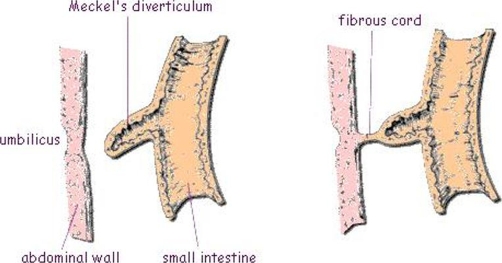
Schematic diagram of MD and the fibrous cord around which small bowel twisted upon itself [9].

The lifetime risk of complications developing in a case of MD is estimated at 4%. Most cases are asymptomatic (80% to 95%). Complications include painless lower gastrointestinal bleeding (4%), intestinal obstruction (6%) secondary to intussusceptions, volvulus, herniation, or entrapment of a loop of bowel through a defect in the diverticular mesentery, around a fibrous band (Figure 3), entrapping an ileal loop within a mesodiverticular band, incarceration within a hernia sac, chronic Meckel’s diverticulitis, foreign body, or neoplasm. Meckel's diverticulitis mimics acute appendicitis (5%). Rarely primary tumor may arise from diverticulum (carcinoid, sarcoma, leiomyoma, adenocarcinoma) [3]. Complicated MD requiring surgery has significant mortality and morbidity, 1.5% and 7% respectively as shown in the epidemiological survey by Cullen et al [4]. Hence, a school of thought recommends resection of asymptomatic MD found incidentally especially in the following scenarios: younger age at presentation (less than 40 years), diverticula longer than 2 cm, narrow diverticular neck, previous abdominal adhesions or obstructions, diverticula with fibrous bands; suspected ectopic gastric tissue; inflamed, thickened diverticula and any palpable or visual abnormality of the Meckel’s diverticulum. Factors that would discourage the resection include older age at presentation, wide diverticular neck and the absence of other abdominal pathology [1,4].

Computed tomography (CT) has 90-94% sensitivity and 96-100% specificity for the diagnosis of small bowel obstruction and a 40-73% positive predictive value for predicting the cause of the obstruction [5,6].

A helical CT is best suited for evaluation of acute or high-grade small-bowel obstruction, as it is able to detect the presence of high-grade small-bowel obstruction, its level, severity, and cause (in about 70% to 80% of cases); presence of ischemia (in about 85% of cases) and presence of closed-loop obstruction or hernia [7]. Sensitivity of CT in the detection of small bowel obstruction ranges from 78% to 100% for complete or high-grade obstruction. For incomplete obstruction, particularly low-grade or intermittent obstruction, the diagnostic accuracy of CT may not be sufficient [8].

Symptomatic MD is traditionally considered a pediatric disease that is associated with intestinal hemorrhage or perforation and is rarely a consideration in the geriatric population. Many different mechanisms can be responsible for complications due to MD in the adult and geriatric population. Misdiagnosis occurs frequently in this age group because of the poor sensitivity of diagnostic tests, nonspecificity of complaints, and lack of recognition that this anomaly can present in the older age group. Physicians need to be cognizant of the multivariate ways of presentation of this commonly assumed pediatric disease especially when evaluating older age group patients for unexplained acute or intermittent abdominal pain, nausea and vomiting, rectal bleeding, peritonitis, or obstruction.

## Abbreviations

CT: Computed tomography
MD: Meckel’s diverticulum
